# Evaluation of the Efficacy of a Dentifrice Tablet to Prevent Dental Caries: A Microbial Study

**DOI:** 10.3390/dj13050201

**Published:** 2025-04-30

**Authors:** Bennett Tochukwu Amaechi, Kannan Kanthaiah, Rayane Farah, Kelly Yang, Amos Chiedu Obiefuna, Parveez Ahamed Abdul-Azees, Mahalakshmi Vijayaraghavan

**Affiliations:** 1 Department of Comprehensive Dentistry, University of Texas Health San Antonio, San Antonio, TX 78229, USA; kannan@americancollege.edu.in (K.K.); rayane.farah@temple.edu (R.F.); abdulazees@uthscsa.edu (P.A.A.-A.); 2 Department of Biology, University of Texas at San Antonio, San Antonio, TX 78249, USA; kelly.yang@my.utsa.edu; 3 Department of Mathematics and Statistics, University of Maryland Global Campus, San Antonio, TX 78236, USA; amos.obiefuna@faculty.umgc.edu; 4 Department of Molecular and Translational Medicine, Texas Tech Health Science Center, El Paso, TX 79905, USA; mahalakshmi.vijayaraghavan@ttuhsc.edu

**Keywords:** dentifrice tablets, tablet toothpaste, hydroxyapatite, caries prevention, demineralization, dental caries

## Abstract

**Background/Objectives:** Dentifrice tablets are a new over-the-counter dentifrice form that are gaining global interest. The aim of this microbial study was to investigate the effectiveness of a dentifrice tablet (DT) containing nanohydroxyapatite (nHAP) to prevent tooth demineralization. **Methods:** 120 bovine tooth blocks were randomly assigned to four treatment groups (30/group): Nanohydroxyapatite DT (5% nHAP), placebo DT (Placebo), NaF toothpaste (1100 ppm Fluoride) and no-treatment (Control). Blocks were subjected to 7-day demineralization by plaque growth in a mixed-organism Microbial Caries Model. Toothpaste was made into slurry (1 toothpaste:3 water), while DT was thoroughly crushed and homogenized with water (1 tablet:3 water) to slurry. Both slurries were applied twice daily for 2 min on each occasion. Demineralization was assessed using Surface Microhardness (SMH) testing before and after plaque exposure. Change (ΔSMH) and percentage change (%∆SMH) in SMH (percentage demineralization [%Dem]), and % demineralization inhibition (%Dem-Inhibition) in each group were calculated. Intra-group (SMH) comparison (paired *t*-test) and intergroup (%∆SMH) comparison (ANOVA/Tukey’s test) were conducted (α = 0.05). **Results:** The paired *t*-test indicated a significant difference (*p* < 0.001) between pre-treatment and post-treatment SMH in all groups. The intergroup comparison based on their %Dem using ANOVA/Tukey’s test showed that Control (29.93 ± 5.58) had significantly (*p* < 0.05) higher %Dem than Placebo (22.81 ± 7.47, *p* < 0.05), nHAP (13.93 ± 11.31, *p* < 0.001) and Fluoride (14.44 ± 10.65, *p* < 0.001). The Placebo had significantly (*p* < 0.01) higher %Dem than nHAP and Fluoride. No significant difference between nHAP and Fluoride. Intergroup comparison based on their %Dem-Inhibition (calculated relative to the control) using ANOVA/Tukey’s test, nHAP (51.74 ± 40.05, *p* < 0.01) and Fluoride (50.56 ± 37.21, *p* < 0.05) had significantly higher %Dem-Inhibition than Placebo tablet (21.86 ± 5.55). No significant difference in %Dem-Inhibition between nHAP and Fluoride. **Conclusions:** The present study demonstrated that dentifrice tablets containing 5% nanohydroxyapatite are as effective as NaF toothpastes containing 1100 ppm fluoride in preventing tooth demineralization.

## 1. Introduction

Dental caries, one of the most common chronic diseases worldwide, results from the loss of minerals from the hard dental tissues caused by the acids that form when the bacteria in the biofilm metabolize dietary carbohydrates [1,2,3,4]. However, carious tissues do not simply undergo constant and cumulative mineral loss, rather they alternate between periods of mineral gain or remineralization, and periods of mineral loss or demineralization [5,6]. Consequently, whether or not the balance between protective factors promoting remineralization (and/or inhibiting demineralization) and pathological factors promoting demineralization tips in one direction, determines if the caries lesion progresses or reverses [2,5]. The restoration of decayed teeth are costly and time consuming, hence, preventive measures are preferred and prioritized [3].

Multiple preventive approaches can be used concomitantly to protect tooth against the occurrence of dental caries [7]. These approaches include the adoption of a low sugar diet, mechanical control of the biofilm through toothbrushing, chemical control of the biofilm through antibacterial agents, inhibition of the demineralization process and promotion of the remineralization process through active agents present in topical oral hygiene products [8,9,10,11].

Fluoride compounds have the most evidence supporting their effectiveness for caries prevention and are commonly incorporated in toothpaste formulations [8,9,10,11,12,13]. However, exposure to considerable amounts of fluoride through prolonged unintentional ingestion is associated with undesirable dental side effects such as fluorosis [14,15,16,17]. The use of toothpaste formulations with low concentrations of fluoride (<1000 ppm) reduces the risk of such effects, but also reduces the protective anti-caries efficacy of fluoride toothpastes [6,15]. Thus, the evaluation of alternative and/or adjunctive, non-fluoride, remineralizing agents became the subject of many recent studies.

Among the non-fluoride alternatives that have been studied, hydroxyapatite (HAP) is one of the most favored. Hydroxyapatite crystals are calcium-phosphate minerals that constitute most of the crystals found in the mineralized tissues of the human body [18,19]. Synthetic HAP crystals are biomimetic particles that have similar physical and chemical properties to the apatite found in the enamel and dentin layers [20,21,22]. Additionally, they have excellent biocompatibility and can be used in reasonable and effective doses, without any risk to the patient’s health, which makes them suitable for use in all age groups [23,24]. Because of these distinctive characteristics, HAP particles are now used in oral hygiene products such as toothpaste, mouth rinses, gels and lotions with a plethora of indications [22,25,26]. Studies comparing HAP and fluoride toothpastes found that both products had comparable effects in caries prevention [27,28,29,30] and remineralization of initial caries lesions [27,28,31]. Other dental applications of HAP are also well-documented and encompass biofilm management [26,32], reduction in hypersensitivity [33], protection against erosion [34,35] and tooth whitening [36,37].

Dentifrice tablets are a new over-the-counter dentifrice form that are gaining global interest. At the moment, there is no existing literature on the considerations for using tablet-based toothpaste as opposed to traditional tube toothpaste. However, collective reports from the manufacturers indicated that toothpaste tablets offer several advantages over traditional tube toothpaste, including reduced plastic waste, portability, a longer shelf life and resistance to heat. Being waterless, toothpaste tablets often do not require chemical preservatives found in liquid toothpaste. They also provide a more environmentally friendly option with minimal or plastic-free packaging, further reducing the environmental impact. Toothpaste tablets do not produce excessive foam, potentially leading to reduced water usage during brushing. Each tablet provides a pre-measured amount of toothpaste, eliminating the controversy over the recommended size of toothpaste for effective oral hygiene. Toothpaste tablets are compact and solid, making them ideal for travel, without the risk of spills or liquid restrictions. The aim of this microbial study was to investigate the effectiveness of dentifrice tablets (Biöm^®^ NOBS™ toothpaste tablets; BIOM LLC, Sheridan, WY, USA) containing nanohydroxyapatite (nanoHAP) to prevent caries development, compared with standard sodium fluoride toothpaste containing 1100 ppm fluoride (Sensodyne^®^; GSK Consumer Healthcare, Warren, NJ, USA) and a placebo dentifrice tablet (Biöm^®^ NOBS™ toothpaste tablets; BIOM LLC, Sheridan, WY, USA). Our null hypothesis was that dentifrice tablets containing nanoHAP would not differ significantly in inhibiting caries development from standard fluoride toothpaste or dentifrice tablets with neither nanoHAP nor fluoride.

## 2. Materials and Methods

Preparation of teeth: Sound bovine teeth were collected and sterilized in accordance with the university procedure. Following sterilization, the teeth were brushed with pumice slurry using a Braun Oral-B Plaque Remover 3D electric toothbrush and then examined by transillumination. Teeth without cracks, hypoplasia, white spot lesions and other malformations were selected. Using a water-cooled diamond wire saw (WELL (Walter Ebner Le Locle) Diamond Wire Saws SA, Le Locle, Switzerland), the roots of each tooth was cut off and tooth blocks (approximately 5 mm length × 5 mm width × 2 mm thick) were produced from the buccal surface of each tooth. A total of 120 tooth blocks were produced. Using adhesive-back lapping film (30 µm) in a MultiPrep™ Precision Polishing machine (Allied High Tech, Cerritos, CA, USA), the enamel surface and the bottom of each block were polished to achieve flat and plano-parallel surfaces required for surface microhardness (SMH) measurement. Following this, all surfaces of each block were painted with two coats of acid-resistant nail varnish, except on the enamel surface.

Measurement of Baseline Surface Microhardness (SMH): The baseline surface microhardness (SMH_b_) of the tooth blocks were measured on each selected tooth block using a Vicker’s diamond indenter (Tukon 2100; Wilson-Instron, Norwood, MA, USA), with a load of 50 g applied for 5 s. The measurement was made at the exposed enamel window (2 mm diameter). Three indentations were made at the middle, upper and lower ends of the enamel surface (preserving a reasonable sound area between the indentations), and the Vicker’s numbers were calculated and averaged for each block.

Study Groupings: Each of the selected 120 blocks was randomly assigned to one of the following four experimental groups, 30 block/group: (A) no treatment, (B) NanoHAP dentifrice tablets (Biöm^®^ NOBS™ toothpaste tablets; BIOM LLC, Sheridan, WY, USA), (C) Fluoride toothpaste (Sensodyne; GSK Consumer Healthcare, Warren, NJ, USA) and (D) Placebo dentifrice tablets without nanoHAP (Biöm^®^ NOBS™ toothpaste tablets; BIOM LLC, Sheridan, WY, USA). The compositions of the products are shown in Table 1. Slurry of the fluoride toothpaste was prepared by mixing 1 part toothpaste (g weight) and 3 parts distilled de-ionized water (DDW) in volume using a laboratory stand mixer until homogenous. The dentifrice tablets were made into slurry by dissolving the tablets in DDW at an appropriate ratio to produce slurry with consistency to that of fluoride toothpaste. Tooth blocks allocation to groups were based on their SMHb such that the values of the mean SMHb for the four groups should not differ significantly. Following grouping, the four groups were subjected to demineralization by plaque growth in our validated Microbial Caries Model (MCM) functioning as an “artificial mouth” as described by Amaechi et al. [38], to test the ability of the dentifrices to inhibit the formation of early caries lesions in bovine tooth enamel.

Experimental procedure (Table 2): The MCM is a multiple-chamber continuous flow culture system described in our previous publication [38]. The four experimental groups were randomly assigned to four culture chambers in the system (30 blocks/chamber). Using heavy duty putty, the tooth blocks were embedded in the vertical grooves on the surface of the cylindrical rod in the culture chamber. The blocks were embedded such that their surfaces flushed with the surface of the cylinder to permit streamlined flow of fluids, and the exposed enamel was available for plaque growth and subsequent demineralization. As previously described [38], the system was operated by continuous circulation of Todd Hewitt Broth (THB) separately through the four chambers to simulate saliva, and 10% sucrose were supplied three times daily for 6 min on each occasion to simulate meals and pH cycling. The pH of plaque in each chamber was monitored at non-feeding time to check the maintenance of neutrality by CO_2_. On the first day of the experiment, pasteurized human whole saliva was circulated through the chamber for 30 min to initiate acquired salivary pellicle formation that laid the foundation for dental plaque growth on tooth blocks. Following pellicle formation, bacterial plaque growth and caries development on the tooth blocks were initiated by circulation of THB inoculated with mixed *Streptococcus mutans* (NCTC 10449, ATCC, Manassas, VA, USA) and *Lactobacilli casei* (NCIB 8820, ATCC, Manassas, VA, USA) culture (broth to inoculum ratio 10:1) for 12 h (adhesion phase). Then, bacteria-free broth was circulated for the rest 12 h of the first day. From day 2, the plaque-covered tooth blocks were treated as shown in Table 2 below and briefly as follows. While experimental group A received no treatment (Control), groups B through D were treated with their respective dentifrice slurry twice daily (morning and evening) for 2 min on each occasion as follows. The cylindrical rod bearing the tooth blocks were immersed into 150 mL of the slurry for 2 min, and then gently rinsed with sterile Phosphate-Buffer Saline (PBS). Fresh slurry of each toothpaste sample was prepared just prior to each treatment episode, and the pH of the toothpaste slurry was measured before treatment. All treatments were carried out inside the incubator at 37 °C and under aseptic condition. The experiment lasted for 5 days.

Post-Treatment SMH Measurement: On termination of the experiment, the tooth blocks were harvested and processed for demineralization assessment by measuring the post-treatment Surface Microhardness (SMH_T_). The SMH_T_ measurement was performed as described above for the baseline measurement by three indentations on the free (un-indented) surface of the block, and the average value was calculated for each block. At this point, the pre-treatment (SMH_b_) and post-treatment (SMH_T_) surface microhardness values of the lesions were available for data analysis.

Sample Size Justification: The gpower software (v.3.1.9.7) was used to determine the sample size needed for the analysis of variance with four equal groups. With a medium effect size of 0.35, an alpha value of 0.05 and a power of 0.85, it was determined that a minimum total sample size of 108 or 27 for each of the four groups was needed. However, to make provisions for possible loss during processing, we used 30 samples per group.

Data Management: SPSS Version 29 was used for all analyses. For all statistical tests, *p* < 0.05 were considered significant. The normality assumption was tested using the Kolmogorov–Smirnov test and non-significance was found for each of the four groups (*p* > 0.05), indicating that the normality assumption was met. The symmetrical shapes of the histograms, as well as the Q-Q plots also indicated normality. The assumptions of equality of variances and normal distribution of errors were tested for the response variables using the histogram, Q-Q plot and the Shapiro–Wilk’s test from the tests of normality table and all confirmed that the normality assumption was met for each variable at the alpha level of α = 0.05.

The mean (n = 30) values of the SMH_b_ and SMH_T_ were calculated for each treatment group and were compared using paired *t*-test to determine if there was any significant change (demineralization) in SMH within each group. To compare the amount of demineralization among the four experimental groups, the percentage change in SMH (%ΔSMH), calculated relative to the baseline (SMH_b_), were determined for each group. Percentage change was used for comparison to make provision for the fact that the tooth blocks in all groups came from different teeth and as such the SMH for the blocks may differ at baseline. This was calculated; thus, %ΔSMH = [(SMH_b_ − SMH_T_)/SMH_b_] × 100. Using the mean values of the %ΔSMH, the four experimental groups were compared among themselves using ANOVA followed by Tukey’s test.

To test our null hypothesis, we had to determine whether the dentifrice groups would not differ significantly in inhibiting caries development, and the percentage demineralization inhibitions by each dentifrice product were calculated relative to the Control (no treatment) group.

## 3. Results

**Figure 1:**
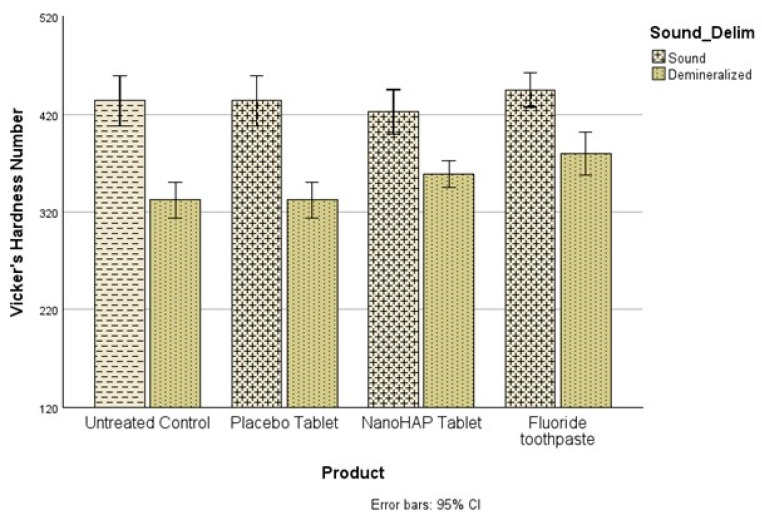
Comparing Vicker’s hardness number (VHN) between before and after demineralization within each treatment. The mean VHN before and after demineralization differed significantly (*p* < 0.001) within all groups.

The pH of the dentifrice slurries measured before treatments were NanoHAP dentifrice 7.02 ± 0.10, Fluoride dentifrice 6.88 ± 0.03 and Placebo dentifrice 7.04 ± 0.05. For the demineralization data, three separate analyses were conducted using SPSS version 28 to address the three objectives of this study. The first objective was to determine whether there were significant differences in Vicker’s hardness number (VHN) between the sound and demineralized teeth (Demineralization). The comparisons were made within each of the four experimental groups, and using paired samples *t*-test, VHN was found to differ significantly (*p* < 0.001) between sound and demineralized teeth within all groups (Figure 1). All paired comparisons had large effect sizes (Cohen’s d), 4.24 (untreated control), 2.38 (Placebo tablet), 1.21 (NanoHAP tablet) and 1.29 (Fluoride toothpaste).

**Figure 2:**
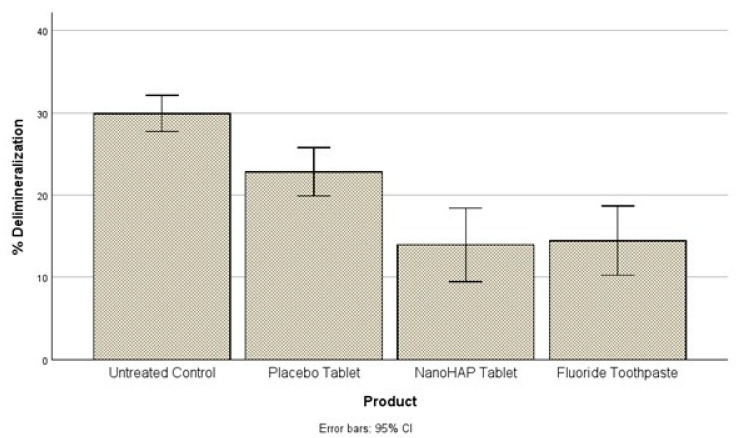
Comparing the treatment groups based on the percentage demineralization that occurred in each group. While the Control group had significantly higher % demineralization than all the dentifrice groups, the Placebo tablet had a significantly higher % demineralization than the NanoHAP tablet and Fluoride toothpaste. No difference between the NanoHAP and Fluoride dentifrices.

The second objective was to compare the percentage demineralization for each of the four experimental groups. For this purpose, the ANOVA was conducted to determine whether statistically significant differences occurred. A statistically significant difference in percentage demineralization among the four groups was observed at the alpha level of 0.05, F(3, 104) = 19.07, *p* < 0.001 (Figure 2). A posthoc analysis showed that the Untreated Control (29.93 ± 5.58) had a higher percentage demineralization than the Placebo tablet (22.81 ± 7.47), NanoHAP tablet (13.93 ± 11.31) and Fluoride toothpaste (14.44 ± 10.65). The differences were statistically significant at the alpha level of 0.05. Placebo tablet had a statistically significant higher percentage demineralization than NanoHAP tablet and Fluoride toothpaste. There was no significant difference between the NanoHAP tablet and the Fluoride toothpaste.

**Figure 3:**
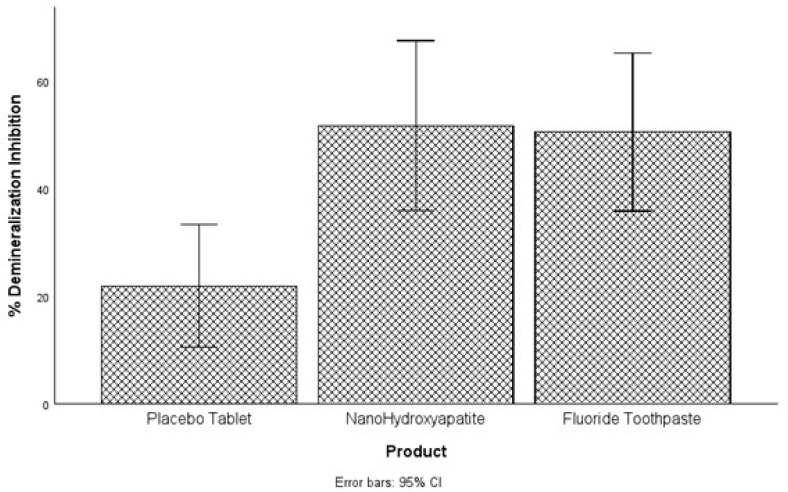
Comparing the treatment groups based on the percentage demineralization inhibition achieved in each group relative to the Control group. While the NanoHAP tablet and Fluoride toothpaste had significantly higher % demineralization inhibition than the Placebo tablet, there was no significant difference between NanoHAP tablet and Fluoride toothpaste.

The last analysis focused on comparing the dentifrices based on their percentage demineralization inhibition. A one-way variance analysis was used, and it was observed that a statistically significant difference in percentage demineralization inhibition exists among the three groups *F*(2, 78) = 6.06, *p* = 0.004, at the alpha level of 0.05 (Figure 3). Tukey test was conducted to compare the percent demineralization inhibition for each pair of the products for significance. NanoHAP tablet (51.74 ± 40.05) and Fluoride toothpaste (50.56 ± 37.21) had statistically significant higher percent demineralization inhibition than Placebo tablet (21.86 ± 5.55), at the alpha level of 0.05. There was no statistically significant difference between NanoHAP tablet and Fluoride toothpaste regarding percent demineralization inhibition.

## 4. Discussion

Toothpaste tablets are a new over-the-counter dentifrice form that are gaining global interest due to several reported advantages it has over the traditional tube toothpaste, such as reduced plastic waste, portability, a longer shelf life, resistance to heat, absence of chemical preservatives and elimination of the controversy over the recommended size of toothpaste for effective oral hygiene. Popular among these commercially available toothpaste tablets are the nanoHAP-containing dentifrice tablets due to the vast evidence supporting the effectiveness of nanoHAP in preventing dental caries and the demonstrated comparable effectiveness with fluoride in caries prevention [27,28,29,30]. However, there has not been any study investigating the effectiveness of these nanoHAP dentifrice tablets in preventing caries. For this reason, we took the lead to investigate the effectiveness of Biöm^®^ NOBS™ toothpaste tablets (BIOM LLC, 30 N Gould St, Sheridan, WY 82801) containing 5% nanHAP in preventing caries development, comparing it with standard sodium fluoride toothpaste containing 1100 ppm fluoride and a placebo dentifrice tablet. The study was conducted using a mixed-species Microbial Caries Model (MCM), produces cariogenic dental plaque and simulates the biological and physiological activities observed within the oral environment [38]. Our MCM has been validated and used in several other studies [39,40]. The MCM produces a well-established early caries lesion (induces demineralization) within 3 days [38], but we extended our study for 5 days to give more time for demineralization considering that interventions were used in some groups, and as such, may delay demineralization in those groups. In the present study, the application of the toothpaste in the presence of plaque, frequently fed with sucrose without toothbrushing, subjected the tooth blocks to the natural demineralization and remineralization cycles similar to a high caries risk condition in the oral environment [38].

It was not surprising in the present study that despite the intervention of the dentifrices, the tooth samples were demineralized (early dental caries) to varied percentages in the three dentifrice groups (Figure 1 and Figure 2). This can be attributed to the fact that in the Artificial Mouth system used in conducting this study, the biofilm was fed with 10% sucrose three times daily without toothbrushing, thus simulating a high caries risk condition precipitated by poor oral hygiene with frequent intake of fermentable carbohydrate, which in real life is a typical condition associated with rampant caries. However, despite the high cariogenic condition, the two active dentifrices inhibited tooth demineralization to high percentages (Figure 3): 5% NanoHAP tablet (51.74 ± 40.05%) and 1100 ppm Fluoride toothpaste (50.56 ± 37.21). The inhibition of demineralization by the nanoHAP dentifrice tablet can be attributed to the various components (Table 1) of this particular dentifrice tablets (Biöm^®^ NOBS™ toothpaste tablets). The main active ingredient in this tablet is nanoHAP, which has been demonstrated in several studies to be effective in caries prevention, remineralization of initial caries lesion and biofilm management [25,27,28,29,30,31,32], due to its distinctive characteristics in relation to natural tooth HAP. When incorporated into oral hygiene products such as toothpastes, mouth rinses, gels and lotions, nanoHAP has been shown to be highly biocompatible with natural tooth HAP, bioactive in function, and has biomimetic mode of actions [18,19,20,24,40]. With regard to caries prevention investigated in the present study, previous studies have reported that nanoHAP can prevent caries development by several mechanisms [20,22,24]. It has been demonstrated that on application of nanoHAP-containing oral hygiene products, the HAP particles strongly adhered to the tooth surface as well as deposit in plaque, and adsorption of bacteria by these HAP crystals induces coaggregation of the bacterial cells, leading to their removal as well as reduce the initial bacterial adherence to the tooth surfaces, thereby reducing biofilm formation and growth [24,25,32,41]. It has also been reported that the deposits of nanoHAP particles in plaque act as a calcium and phosphate ions reservoir, which are released when plaque pH becomes acidic, and these ions act as a buffering solution to neutralize acids, thus reducing the demineralization potential of the plaque as well as promoting remineralization [5,24,42,43]. Furthermore, HAP in oral care products, such as toothpaste, has been shown to elevate calcium and phosphate ions concentrations in saliva and plaque, thus maintaining a topical state of supersaturation of these ions with respect to tooth minerals, thereby inhibiting demineralization and enhances remineralization [6]. Another caries preventive active ingredient in this dentifrice tablet is Xylitol, which has long been established to prevent tooth demineralization [44,45,46,47,48]. Xylitol inhibits the metabolism of sucrose by plaque cariogenic bacteria by forming xylitol-5-phosphate, which inhibits glycolytic enzymes, thereby inhibiting bacterial growth and acid production [46,47,48]. It is most effective against *Streptococcus mutans*, the primary bacteria responsible for the dental caries process, [14] which was included in the Artificial Mouth system used in the present study. Furthermore, the presence of xylitol-5-phosphate results in the failure of cariogenic bacteria to adhere to dental surfaces [48]. Other ingredients of the dentifrice tablets that has been proven to have antimicrobial effect on plaque bacterial and inhibits its growth and acid production are Zinc Citrate [49,50] as well as Hydrated silica, Calcium carbonate and menthol [51]. Calcium carbonate creates a hostile environment against the growth of aciduric bacteria by increasing the pH in plaque [51]. Menthol is thought to have antibacterial activity by altering the bacterial cell wall, and dentifrices that contains this active ingredient have been reported to reduce plaque significantly [51].

As one should expect, considering its content of calcium carbonate (Table 1), the Placebo dentifrice tablet also inhibited demineralization to some extent (21.86 ± 5.55%). Calcium carbonate, as stated above, provides bicarbonate ions that increases the pH in plaque, and in this way, neutralizing acids produced by plaque bacteria as well as creating a hostile environment against the growth of aciduric cariogenic bacteria [51]. Furthermore, calcium carbonate serves as a source of Ca+ ions, which would saturate the plaque and as such, reduces enamel solubility and increases remineralization of enamel [51]. These distinctive characteristics of calcium carbonate enable it to inhibit demineralization and prevent caries. However, its demineralization inhibition was almost 2.5 times less than that of NanoHAP tablet and Fluoride toothpaste, obviously due to the absence of mainly the active ingredients, nanoHAP or fluoride and other caries preventive agents (Zinc Citrate, Hydrated silica and menthol) as contained in the nanoHAP and fluoride dentifrices (Table 1). One may argue that the calcium carbonate content of the placebo tablet may potentially underestimate the true efficacy of nHAP. However, because the tested marketed nHAP dentifrice tablet contains calcium carbonate, we decided to also incorporate calcium carbonate into the placebo to enable the demineralization inhibition effect of the active ingredient (nHAP) to be observed and to prevent the nHAP tablet having double advantage over the placebo tablet with regard to demineralization inhibition.

The equal effectiveness (statistically non-significant difference) of fluoride toothpaste and nanoHAP dentifrice tablets in inhibiting tooth demineralization as observed in the present study agrees with the report of previous studies [27,28,29,30,31]. Studies comparing HAP and fluoride toothpastes found that both products had comparable effects in caries prevention [27,29,30,31,52] and remineralization of initial caries lesions [27,28,31]. It may be surprising that the inhibition of demineralization by fluoride in the present study is limited to approximately 51%. It is well established that even though fluoride can still penetrate plaque to reach the tooth surface, its ability to protect the tooth against decay is diminished in the presence of a large amount of plaque. This is because it would not fully counteract the high levels of acid produced by the plaque buildup in a frequent sucrose exposure as applied in the Artificial Mouth used in the present study [53,54,55]. It is pertinent to mention that the pH range of the dentifrice slurries, 7.02 ± 0.10 (nHAP dentifrice), 6.88 ± 0.03 (Fluoride dentifrice) and 7.04 ± 0.05 (Placebo dentifrice) are considered neutral pH and as such may not possibly influence bacterial activity or demineralization rates (SMH outcomes). If even it did, it equally affected all three products, and as such, did not influence the differences in the outcomes.

Although the Artificial Mouth used in the present study mimicked the biological activities of the oral environment as closely as possible, there are still some limitations, one of which is being an in vitro study where many confounding variables that would be encountered in the oral cavity were controlled. However, the findings garnered from the present in vitro study can be used as a foundation for developing further studies aimed at testing the dentifrice tablet in clinical trials to confirm its effectiveness in preventing caries. The clinical trial, which would obviously involve brushing, will take care of the gap (lack of brushing simulation) in the present in vitro studies.

## 5. Conclusions

Within the limits of this in vitro study, the tested dentifrice tablet (Biöm^®^ NOBS™ toothpaste tablets) containing 5% nanohydroxyapatite showed a significant ability to inhibit tooth surface demineralization, which is comparable to that of toothpaste containing 1100 ppm of fluoride. Thus, nanohydroxyapatite toothpaste tablet can serve as an effective alternative to over-the-counter standard fluoride toothpaste. It further demonstrated that the tested toothpaste tablet can inhibit dental caries development amid heavy dental plaque.

## Figures and Tables

**Table 1:** Study products and their compositions.

Product	Company	Composition
NanoHAP dentifrice tablets	BIOM LLC, 30 N Gould St, #40371, Sheridan, WY, USA	5% Nanohydroxyapatite, Xylitol, Hydrated silica, Sodium Carbonate, Calcium Carbonate, Zinc Citrate, Sodium Cocoyl Isethionate, Peppermint, Menthol, Cellulose, Surfactant from coconut oil, Licorice Root Extract and Xanthan gum.
Placebo dentifrice tablet	BIOM LLC, 30 N Gould St, #40371, Sheridan, WY, USA	Saccharose, Sucralose, Microcrystalline Cellulose, Calcium Carbonate, Sodium Cocoil Isethionate, Xanthan gum, Natural mint flavor.
Sensodyne antisensitivity and anticavity toothpaste	GSK Consumer Healthcare, Warren, NJ, USA	0.25% (0.15% W/V Fluoride Ion) Sodium fluoride, 5% Potasium nitrate, Sorbitol, water, Hydrated Silica, Glycerin, Cocamidopropyl betaine, flavor, Xanthan gum, Titanium Dioxide, Sodium Saccharin, Sodium hydroxide, Sucralose, Yellow 10, Blue 1.

**Table 2:** Treatment Schedule for the Microbial Caries Model for this study.

Day	Time	Treatment
Day 1	30 min	Acquired salivary pellicle formation.
	First 12 h	Bacteria-inoculated Todd Hewitt Broth (THB) was circulated for 12 h (adhesion phase).
	Second 12 h	Circulation of bacteria-free THB.
Day 2–Day 5	7:00	Toothpaste (2 min) treatment.
	7:02	Circulation of bacteria-free THB re-starts.
	8:00	Sucrose circulation for 6 min.
	8:06	Circulation of bacteria-free THB re-starts.
	12:00	Sucrose circulation for 6 min.
	12:06	Circulation of bacteria-free THB re-starts.
	16:00	Toothpaste (2 min) treatment.
	16:02	Circulation of bacteria-free Todd Hewitt Broth (THB) re-starts.
	17:00	Sucrose circulation for 6 min.
	17:06 until 7:00 am next day	Circulation of bacteria-free THB re-starts.

## Data Availability

The data presented in this study are available upon reasonable request from the corresponding author (B.T.A.).

## References

[1] James S.L., Abate D., Abate K.H., Abay S.M., Abbafati C., Abbasi N., Abbastabar H., Abd-Allah F., Abdela J., Abdelalim A. (2018). Global, regional, and national incidence, prevalence, and years lived with disability for 354 diseases and injuries for 195 countries and territories, 1990–2017: A systematic analysis for the Global Burden of Disease Study 2017. Lancet.

[2] Featherstone J., Chaffee B. (2018). The evidence for caries management by risk assessment (CAMBRA^®^). Adv. Dent. Res..

[3] Lawson N.C. (2025). Current Evidence for Caries Prevention and Enamel Remineralization. Compend. Contin. Educ. Dent. (15488578).

[4] Simón-Soro A., Mira A. (2015). Solving the etiology of dental caries. Trends Microbiol..

[5] Philip N. (2019). State of the art enamel remineralization systems: The next frontier in caries management. Caries Res..

[6] Cieplik F., Rupp C.M., Hirsch S., Muehler D., Enax J., Meyer F., Hiller K.-A., Buchalla W. (2020). Ca^2+^ release and buffering effects of synthetic hydroxyapatite following bacterial acid challenge. BMC Oral Health.

[7] Fejerskov O., Nyvad B., Kidd E. (2015). Dental Caries: The Disease and Its Clinical Management.

[8] Walsh T., Worthington H.V., Glenny A.M., Marinho V.C., Jeroncic A. (2019). Fluoride toothpastes of different concentrations for preventing dental caries. Cochrane Database Syst. Rev..

[9] Wierichs R., Zelck H., Doerfer C., Appel P., Paris S., Esteves-Oliveira M., Meyer-Lückel H. (2017). Effects of dentifrices differing in fluoride compounds on artificial enamel caries lesions in vitro. Odontology.

[10] Moynihan P., Kelly S. (2014). Effect on caries of restricting sugars intake: Systematic review to inform WHO guidelines. J. Dent. Res..

[11] Nobre C.M., König B., Pütz N., Hannig M. (2021). Hydroxyapatite-based solution as adjunct treatment for biofilm management: An in situ study. Nanomaterials.

[12] Marinho V.C., Chong L.Y., Worthington H.V., Walsh T. (2016). Fluoride toothpastes for preventing dental caries in children and adolescents. Cochrane Database Syst Rev..

[13] Wong M., Clarkson J., Glenny A.-M., Lo E., Marinho V., Tsang B., Walsh T., Worthington H. (2011). Cochrane reviews on the benefits/risks of fluoride toothpastes. J. Dent. Res..

[14] Ekambaram M., Itthagarun A., King N.M. (2011). Ingestion of fluoride from dentifrices by young children and fluorosis of the teeth-a literature review. J. Clin. Pediatr. Dent..

[15] Green R., Lanphear B., Hornung R., Flora D., Martinez-Mier E.A., Neufeld R., Ayotte P., Muckle G., Till C. (2019). Association between maternal fluoride exposure during pregnancy and IQ scores in offspring in Canada. JAMA Pediatr..

[16] Farmus L., Till C., Green R., Hornung R., Mier E.A.M., Ayotte P., Muckle G., Lanphear B.P., Flora D.B. (2021). Critical windows of fluoride neurotoxicity in Canadian children. Environ. Res..

[17] Zohoori F., Maguire A. (2018). Are there good reasons for fluoride labelling of food and drink?. Br. Dent. J..

[18] Enax J., Epple M. (2018). Synthetic hydroxyapatite as a biomimetic oral care agent. Oral Health Prev. Dent..

[19] Limeback H., Enax J., Meyer F. (2021). Biomimetic hydroxyapatite and caries prevention: A systematic review and meta-analysis. Can. J. Dent. Hyg..

[20] Nobre C.M.G., Pütz N., Hannig M. (2020). Adhesion of hydroxyapatite nanoparticles to dental materials under oral conditions. Scanning.

[21] Carella F., Degli Esposti L., Adamiano A., Iafisco M. (2021). The use of calcium phosphates in cosmetics, state of the art and future perspectives. Materials.

[22] Chen L., Al-Bayatee S., Khurshid Z., Shavandi A., Brunton P., Ratnayake J. (2021). Hydroxyapatite in oral care products—A review. Materials.

[23] Epple M. (2018). Review of potential health risks associated with nanoscopic calcium phosphate. Acta Biomater..

[24] Enax J., Fabritius H.-O., Fabritius-Vilpoux K., Amaechi B.T., Meyer F. (2019). Modes of action and clinical efficacy of particulate hydroxyapatite in preventive oral health care-state of the art. Open Dent. J..

[25] Kensche A., Holder C., Basche S., Tahan N., Hannig C., Hannig M. (2017). Efficacy of a mouthrinse based on hydroxyapatite to reduce initial bacterial colonisation in situ. Arch. Oral Biol..

[26] Sudradjat H., Meyer F., Loza K., Epple M., Enax J. (2020). In vivo effects of a hydroxyapatite-based oral care gel on the calcium and phosphorus levels of dental plaque. Eur. J. Dent..

[27] O’Hagan-Wong K., Enax J., Meyer F., Ganss B. (2022). The use of hydroxyapatite toothpaste to prevent dental caries. Odontology.

[28] Cocco F., Salerno C., Wierichs R.J., Wolf T.G., Arghittu A., Cagetti M.G., Campus G. (2025). Hydroxyapatite-Fluoride Toothpastes on Caries Activity: A Triple-Blind Randomized Clinical Trial. Int. Dent. J..

[29] Grocholewicz K., Matkowska-Cichocka G., Makowiecki P., Droździk A., Ey-Chmielewska H., Dziewulska A., Tomasik M., Trybek G., Janiszewska-Olszowska J. (2020). Effect of nano-hydroxyapatite and ozone on approximal initial caries: A randomized clinical trial. Sci. Rep..

[30] Paszynska E., Pawinska M., Gawriolek M., Kaminska I., Otulakowska-Skrzynska J., Marczuk-Kolada G., Rzatowski S., Sokolowska K., Olszewska A., Schlagenhauf U. (2021). Impact of a toothpaste with microcrystalline hydroxyapatite on the occurrence of early childhood caries: A 1-year randomized clinical trial. Sci. Rep..

[31] Paszynska E., Pawinska M., Enax J., Meyer F., Schulze zur Wiesche E., May T.W., Amaechi B.T., Limeback H., Hernik A., Otulakowska-Skrzynska J. (2023). Caries-preventing effect of a hydroxyapatite-toothpaste in adults: A 18-month double-blinded randomized clinical trial. Front. Public Health.

[32] Meyer F., Enax J. (2019). Hydroxyapatite in oral biofilm management. Eur. J. Dent..

[33] Hu M.-L., Zheng G., Lin H., Yang M., Zhang Y.-D., Han J.-M. (2019). Network meta-analysis on the effect of desensitizing toothpastes on dentine hypersensitivity. J. Dent..

[34] Fabritius-Vilpoux K., Enax J., Mayweg D., Meyer F., Herbig M., Raabe D., Fabritius H.-O. (2021). Ultrastructural changes of bovine tooth surfaces under erosion in presence of biomimetic hydroxyapatite. Bioinspired Biomim. Nanobiomater..

[35] Lelli M., Putignano A., Marchetti M., Foltran I., Mangani F., Procaccini M., Roveri N., Orsini G. (2014). Remineralization and repair of enamel surface by biomimetic Zn-carbonate hydroxyapatite containing toothpaste: A comparative in vivo study. Front. Physiol..

[36] Epple M., Meyer F., Enax J. (2019). A critical review of modern concepts for teeth whitening. Dent. J..

[37] Shang R., Kaisarly D., Kunzelmann K.-H. (2022). Tooth whitening with an experimental toothpaste containing hydroxyapatite nanoparticles. BMC Oral Health.

[38] Amaechi B.T., Abdul Azees P.A., Farah R., Movaghari Pour F., Dillow A.M., Lin C.-Y. (2023). Evaluation of an artificial mouth for dental caries development. Microorganisms.

[39] Karthikeyan R., Amaechi B.T., Rawls H.R., Lee V.A. (2011). Antimicrobial activity of nanoemulsion on cariogenic *Streptococcus mutans*. Arch. Oral Biol..

[40] Ramalingam K., Amaechi B.T., Ralph R.H., Lee V.A. (2012). Antimicrobial activity of nanoemulsion on cariogenic planktonic and biofilm organisms. Arch. Oral Biol..

[41] Fabritius-Vilpoux K., Enax J., Herbig M., Raabe D., Fabritius H.-O. (2019). Quantitative affinity parameters of synthetic hydroxyapatite and enamel surfaces in vitro. Bioinspired Biomim. Nanobiomater..

[42] Hannig M., Hannig C. (2012). Nanotechnology and its role in caries therapy. Adv. Dent. Res..

[43] Schäfer F., Beasley T., Abraham P. (2009). In vivo delivery of fluoride and calcium from toothpaste containing 2% hydroxyapatite. Int. Dent. J..

[44] Sharif M.O., Ahmed F., Worthington H.V. (2013). Xylitol-containing products for preventing dental caries in children and adolescents. Cochrane Database Syst. Rev..

[45] ALHumaid J., Bamashmous M. (2022). Meta-analysis on the effectiveness of xylitol in caries prevention. J. Int. Soc. Prev. Community Dent..

[46] Takahashi N., Washio J. (2011). Metabolomic effects of xylitol and fluoride on plaque biofilm in vivo. J. Dent. Res..

[47] Trahan L. (1995). Xylitol: A review of its action on mutans streptococci and dental plaque--its clinical significance. Int. Dent. J..

[48] Vadeboncoeur C., Trahan L., Mouton C., Mayrand D. (1983). Effect of xylitol on the growth and glycolysis of acidogenic oral bacteria. J. Dent. Res..

[49] Sreenivasan P., Furgang D., Markowitz K., McKiernan M., Tischio-Bereski D., Devizio W., Fine D. (2009). Clinical anti-microbial efficacy of a new zinc citrate dentifrice. Clin. Oral Investig..

[50] Hu D., Sreenivasan P., Zhang Y., De Vizio W. (2010). The effects of a zinc citrate dentifrice on bacteria found on oral surfaces. Oral Health Prev. Dent..

[51] Vranic E., Lacevic A., Mehmedagic A., Uzunovic A. (2004). Formulation ingredients for toothpastes and mouthwashes. Bosn. J. Basic Med. Sci..

[52] Amaechi B.T., AbdulAzees P.A., Alshareif D.O., Shehata M.A., Lima P.P.d.C.S., Abdollahi A., Kalkhorani P.S., Evans V. (2019). Comparative efficacy of a hydroxyapatite and a fluoride toothpaste for prevention and remineralization of dental caries in children. BDJ Open.

[53] Watson P., Pontefract H., Devine D., Shore R., Nattress B., Kirkham J., Robinson C. (2005). Penetration of fluoride into natural plaque biofilms. J. Dent. Res..

[54] Tokura T., Robinson C., Watson P., Abudiak H., Nakano T., Higashi K., Naganawa T., Kato K., Fukuta O., Nakagaki H. (2012). Effect of pH on fluoride penetration into natural human plaque. Pediatr. Dent. J..

[55] Stoodley P., Wefel J., Gieseke A., DeBeer D., Von Ohle C. (2008). Biofilm plaque and hydrodynamic effects on mass transfer, fluoride delivery and caries. J. Am. Dent. Assoc..

